# Cocksfoot mottle virus coat protein is dispensable for the systemic infection

**DOI:** 10.1186/1743-422X-11-19

**Published:** 2014-02-04

**Authors:** Allan Olspert, Kristjan Kamsol, Cecilia Sarmiento, Jelena Gerassimenko, Erkki Truve

**Affiliations:** 1 Department of Gene Technology, Tallinn University of Technology, Akadeemia tee 15, 12618 Tallinn, Estonia; 2 Current address: Department of Plant Sciences, University of Cambridge, Downing Street, Cambridge CB2 3EA, UK

**Keywords:** Sobemovirus, Virus movement, RNA silencing suppressor, Coat protein

## Abstract

**Background:**

The *Sobemovirus* genome consists of polycistronic single-stranded positive-sense RNA. The first ORF encodes P1, a suppressor of RNA silencing required for virus movement. The coat protein (CP) is expressed from the 3′ proximal ORF3 via subgenomic RNA. In addition to its structural role, the CP of some sobemoviruses has been reported to be required for systemic movement and to interact with P1. The aim of this study was to analyse the role of *Cocksfoot mottle virus* (CfMV) CP in the suppression of RNA silencing and virus movement.

**Methods:**

*Agrobacterium*-mediated transient expression method was used for testing CfMV CP capacity to suppress RNA silencing. CP substitution and deletion mutants were generated to examine the role of this protein in CfMV infection, using three host plants (oat, barley and wheat). The viral movement was characterised with CfMV expressing EGFP fused to the C-terminus of CP.

**Results:**

In the current study we show that CfMV CP is an additional RNA silencing suppressor. Interestingly, we observed that all CP mutant viruses were able to infect the three tested host plants systemically, although usually with reduced accumulation. CfMV expressing EGFP was detected in epidermal and mesophyll cells of inoculated leaves. Although EGFP fluorescence was not detected in upper leaves, some plants displayed CfMV symptoms. Analysis of the upper leaves revealed that the viruses had lost the EGFP sequence and sometimes also most of the CP gene.

**Conclusions:**

The present study demonstrates that CfMV CP suppresses RNA silencing but, surprisingly, is dispensable for systemic movement. Thus, CfMV does not move as virion in the tested host plants. The composition of the movement RNP complex remains to be elucidated.

## Background

*Sobemovirus* is a small genus of plant viruses with single-stranded positive-sense RNA genome (for review [1]). Sobemoviruses have a viral protein genome linked (VPg) covalently attached to the 5′ end of genomic and subgenomic RNA. The 5′ proximal open reading frame 1 (ORF1) encodes the P1 protein, while the viral capsid or coat protein (CP) is expressed from the 3′ proximal ORF3 via subgenomic RNA. The central part of the genome encodes the viral polyproteins P2a and P2ab, the latter is translated through a-1 programmed frameshift mechanism [2].

Sobemoviral proteins, and CP among them, are multifunctional. CPs primary function is structural, being the building block for the virion. Virions of sobemoviruses have an icosahedral structure according to T = 3 symmetry comprising of 180 CP monomers [3]. Though the primary sequences of sobemoviral CPs are poorly conserved, their three-dimensional structures are highly similar. Sobemoviral CPs contain two domains–the N-terminal R (random) and C-terminal S (shell) domain which is responsible for subunit-subunit contacts in the virion [3-8]. The N-terminal part of all sobemoviral CPs is rich in basic amino acids and contains an arginine-rich region, which is necessary for CP-RNA interactions and RNA encapsidation [9-11]. It has also been shown that the N-terminal part of *Southern cowpea mosaic virus* (SCPMV) CP interacts with membranes [12] and that the N-terminus of *Cocksfoot mottle virus* (CfMV) CP contains functional nuclear localisation signals [13]. CPs have also been reported to be involved in virus movement. For instance, CPs of *Rice yellow mottle virus* (RYMV) and SCPMV are required for cell-to-cell movement, but are not necessary for virus replication [14,15]. Moreover, long distance movement of RYMV and SCPMV has been proposed to be dependent on viral particle formation [16,17]. The CP of *Sesbania mosaic virus* (SeMV) has been shown to interact with P1, which is a suppressor of RNA silencing and has been implicated in virus movement [18-20].

In this study we analyse the role of CfMV CP in suppression of RNA silencing and virus movement. We show that the CP of CfMV acts as an RNA silencing suppressor and that CP is dispensable for cell-to-cell as well as systemic movement of CfMV in oats, wheat and barley.

## Results

### CP and RNA silencing

Viruses can encode more than one RNA silencing suppressor and the P1 protein has been shown to interact with CP [18]. Therefore we decided to test if CP affects RNA silencing. Using the *Agrobacterium*-mediated transient expression method we infiltrated *N. benthamiana* 16c line, expressing GFP, with *Agrobacterium* carrying the RNA silencing inducer GFP gene together with *Agrobacterium* containing CfMV CP gene. GFP together with the empty vector or with CfMV P1 were used as controls. The CP’s potential influence on the P1 suppression activity was also assessed by infiltrating a mixture of *Agrobacterium* carrying both genes together with GFP.

**Figure 1:**
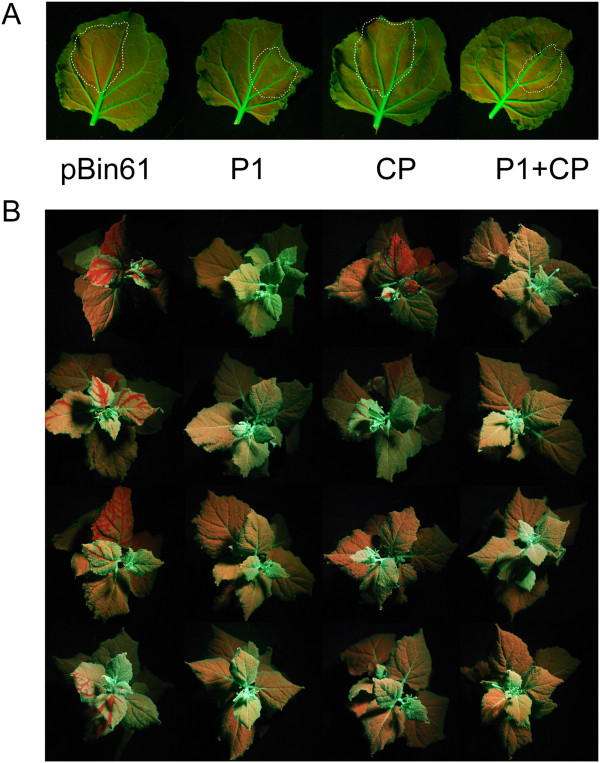
**CP of CfMV is a suppressor of RNA silencing. (A) ***N. benthamiana* 16c leaves infiltrated with *A. tumefaciens* harbouring the constructs shown on the lower part of the panels (empty vector pBin61, P1 of CfMV, CP of CfMV) together with *A. tumefaciens* harbouring GFP. Pictures were taken 7 dpi under UV light. Dotted lines show the border of the infiltrated patches. **(B)***N. benthamiana* 16c plants infiltrated as in **(A)**. Four representative plants are shown for each infiltration. Pictures were taken at 14 dpi under UV light.

At 7 days post-inoculation (dpi) RNA silencing of GFP was clear in the infiltrated patch of leaves inoculated with the empty vector (pBin61). The leaves infiltrated with the *Agrobacterium* carrying the CP showed less GFP silencing (faint red) and the ones infiltrated with P1 or with the mixture containing P1 and CP showed a silenced area only at the border of the patch (Figure 1A). Two weeks after the infiltration the systemic silencing in the upper leaves was seen in 84% of pBin61 inoculated plants, 61% of CP inoculated plants, 6% of P1 inoculated plants and 25% of the plants inoculated with the mixture of P1 and CP. At 28 dpi the percentages declined to 75% in the case of the empty vector and 29% in the case of CP. Plants infiltrated with P1 or with the mixture of P1 plus CP were completely green at the top and in general the systemic silencing of GFP all over the plants was minimal (Figure 1B).

**Figure 2:**
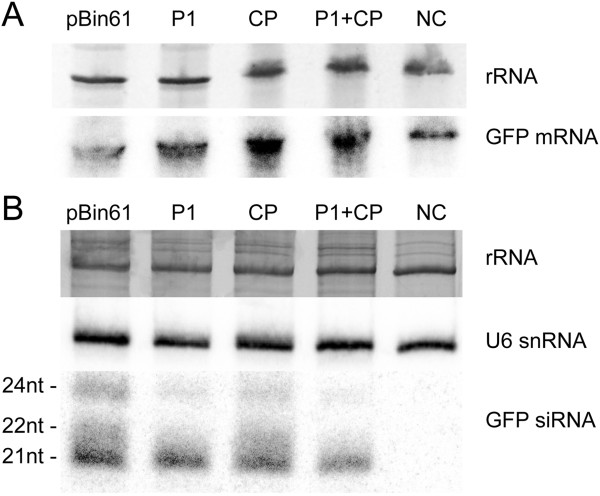
**Northern blot analysis showing the RNA silencing suppressor activity of CfMV CP. (A)** Northern blot of RNA isolated from the infiltrated patches of *N. benthamiana* 16c leaves infiltrated as indicated in Figure 1. The upper part shows ethidium bromide staining of rRNA as loading control. The lower part shows the radioactive detection of GFP mRNA. **(B)** RNA isolated from infiltrated patches of *N. benthamiana* 16c leaves infiltrated as indicated in Figure 1 were used for detecting GFP siRNAs. The upper part shows ethidium bromide staining of rRNA as loading control. The central part shows the radioactive detection of U6 snRNA also as loading control. The lower part shows the radioactive detection of GFP siRNAs (24 nt, 22 nt and 21 nt long). NC: negative control (non-infiltrated *N. benthamiana* 16c).

The molecular analysis of GFP mRNA and siRNAs in the infiltrated patches of these leaves confirmed that CP was a suppressor of RNA silencing. The GFP mRNA levels in the leaf infiltrated with the empty vector pBin61 were lower than the GFP mRNA levels from the other infiltrated leaves. The leaves infiltrated with P1, CP or both, contained enhanced levels of GFP mRNA compared with the GFP mRNA level of a non-infiltrated leaf (NC, Figure 2A). In the case of pBin61 the amount of 21-nt, 22-nt and 24-nt siRNAs was the highest. CP and P1 reduced the amount of siRNAs and the biggest effect was observed in the case of the 24-nt ones. The mixture of P1 and CP produced a strong reduction of siRNAs (Figure 2B).

### Infectivity of CP mutants

**Figure 3:**
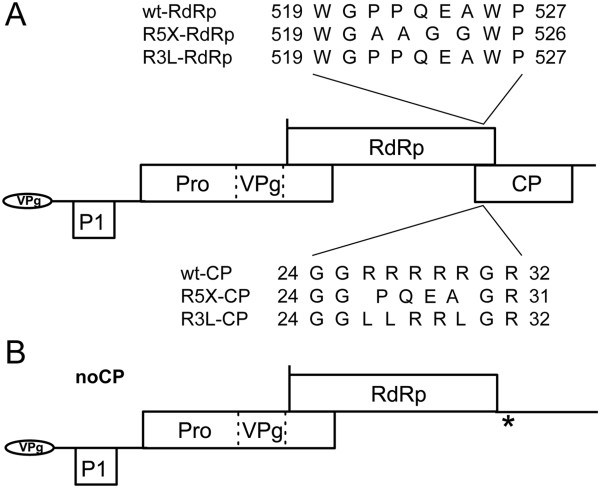
**Overview of the mutations introduced into the CfMV genome. (A)** Mutations in the arginine rich region of CP and the corresponding mutations in RdRp. The RdRp is translated in-1 reading frame in relation to CP. Wild type (wt-RdRp and wt-CP) and mutated (R5X-RdRp, R5X-CP, R3L-RdRp and R3L-CP) amino acid sequences of the changed regions are indicated in single letter code. CfMV genome: Pro, protease domain, VPg, VPg domain. **(B)** Representation of CfMV mutant noCP, in which the initiation codon of CP is mutated and an additional stop codon, indicated with an asterisk, is introduced into the CP reading frame after RdRp sequence.

Since the CP of some sobemoviruses has been reported to be required for virus movement, we examined the role of CP arginine-rich region and CP deletion in viral infection cycle. As the region encoding the arginine-rich region of CfMV CP overlaps with the coding part of RdRp, two different arginine-rich region mutants were generated. In mutant R5X, five arginines were replaced by four non-basic amino acid residues, which also caused the introduction of mutations into the RdRp gene (Figure 3A). In mutant R3L, three out of five arginines were replaced by leucines and no mutations were introduced to RdRp. A full CP knockout virus, titled noCP, was created by mutating the CP initiation codon(s) AUGAUG to ACGACG and by introducing a stop codon into the CP reading frame after its overlap with RdRp gene (Figure 3B).

**Figure 4:**
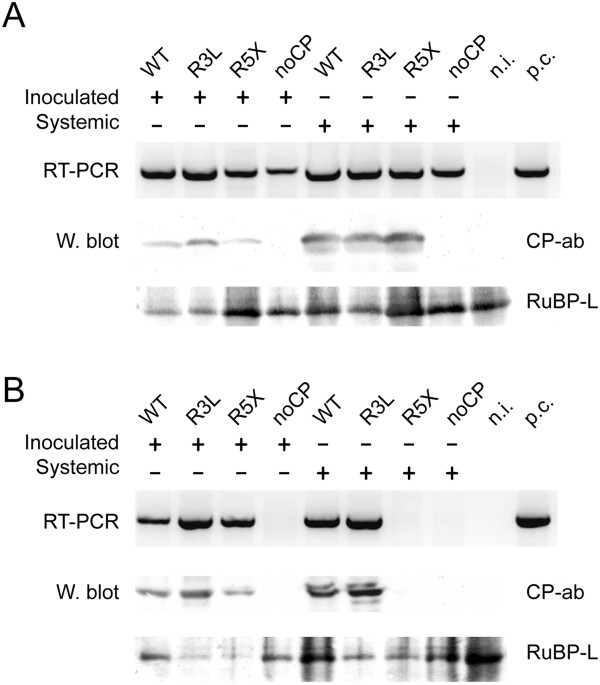
**Detection of CfMV infection in oat plants inoculated with CfMV. (A)** RT-PCR–Reverse transcription PCR analysis of total RNA extracted from plants biolistically inoculated with CfMV (WT) and CfMV mutants (R3L, R5X, noCP). RNA from a non-inoculated plant (n.i.) was used as negative control and RNA from a plant previously known to be infected was used as positive control (p.c.). Samples were collected from the inoculated leaves (Inoculated) at 14 dpi and from upper leaves (Systemic) at 21 dpi. Primers amplifying the region of RdRp and CP genes were used. W.blot–Western blot analysis of plant total protein extracts with polyclonal anti CfMV CP antibody (CP-ab). An additional positive control was not used. Ribulose-1,5-bisphosphate carboxylase oxygenase large subunit (RuBP-L) was visualised with Ponceau S stain as loading control. **(B)** Detection of CfMV infection in sap-inoculated plants. Oat plants were sap-inoculated with CfMV and its mutants. Analysis and annotation as described in **(A)**.

Oat plants were biolistically inoculated with the mutant viruses and tested for infection by RT-PCR and Western blot (Figure 4). The analysis of inoculated leaves revealed that all three mutants were able to replicate and accumulate in the inoculated leaves. To our surprise all three mutants were also able to infect the host plant systemically. Sequencing of the fragments obtained by RT-PCR proved that the viral RNA detected from upper leaves still carried all the mutations (data not shown). As expected we were not able to detect CP in the case of mutant noCP (Figure 4A, W. blot). Indeed, CP was undetectable for this mutant even after enrichment for CP by immunoprecipitation (data not shown).

### Mechanical transmission of CP mutants

As CP is essential for the formation of virus particles, it was interesting to see whether the mutant viruses would be transmissible by mechanical inoculation. Oat plants were inoculated with sap obtained from the upper leaves of plants infected with the mutants and analysed for CfMV infection as described above (Figure 4B). Mechanical transmission of mutant R3L occurred in a similar way to wild type (wt) virus, infection was detected in both inoculated and upper leaves. In the case of mutant R5X the transmission was also successful as the virus was detected in the inoculated leaves, but the mutant was unable to infect the plants systemically. For both mutants the presence of the mutations in the viral RNA was verified by sequencing (data not shown). Interestingly, we were unable to detect infection in plants inoculated with noCP.

### Infectivity of mutant noCP in other hosts

**Figure 5:**
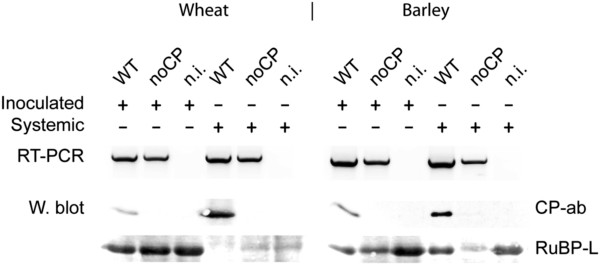
**Detection of CfMV and mutant noCP in biolistically inoculated wheat and barley plants.** The host plant is indicated above the panels. RT-PCR – Reverse transcription PCR analysis of total RNA extracted from plants inoculated with CfMV (WT) and CfMV CP deletion mutant (noCP). Samples from a non-inoculated plant (n.i.) were used as negative control. Samples were collected from the inoculated leaves (Inoculated) at 14 dpi and from upper leaves (Systemic) at 21 dpi. Primers amplifying the region of RdRp and CP genes were used. W.blot–Western blot analysis of plant total protein extracts with polyclonal anti CfMV CP antibody (CP-ab). Ribulose-1,5-bisphosphate carboxylase oxygenase large subunit (RuBP-L) was visualised with Ponceau S stain as loading control.

In order to analyse whether the CP of CfMV is dispensable for infection and systemic movement also in other host plants, wheat and barley plants were biolistically inoculated with CfMV and the mutant noCP. The systemic movement of noCP occurred in both host species similarly to what was observed in oat (Figure 5). In wheat, the mutant noCP produced strong systemic symptoms similarly to wt CfMV (data not shown).

### Movement of CP-EGFP and CPdelta-EGFP viruses

**Figure 6:**
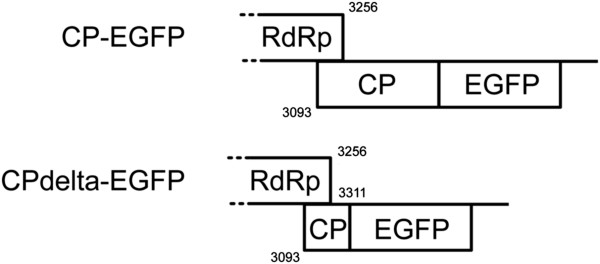
**Schematic representation of EGFP expressing CfMV viruses.** CP-EGFP–EGFP is fused to the C-terminus of full-length CP. CPdelta-EGFP–EGFP is replacing CP C-terminus starting from the nucleotide position 3311. Both viruses have full-length 3′ UTR.

Since previous experiments demonstrated that the CP was dispensable for cell-to-cell and long distance movement of CfMV, it was decided to further characterise CfMV movement. Therefore, two EGFP expressing CfMV viruses were constructed (Figure 6). EGFP was fused to the C-terminus of the full length CP (CP-EGFP) or placed instead of the CP C-terminus (CPdelta-EGFP).

**Figure 7:**
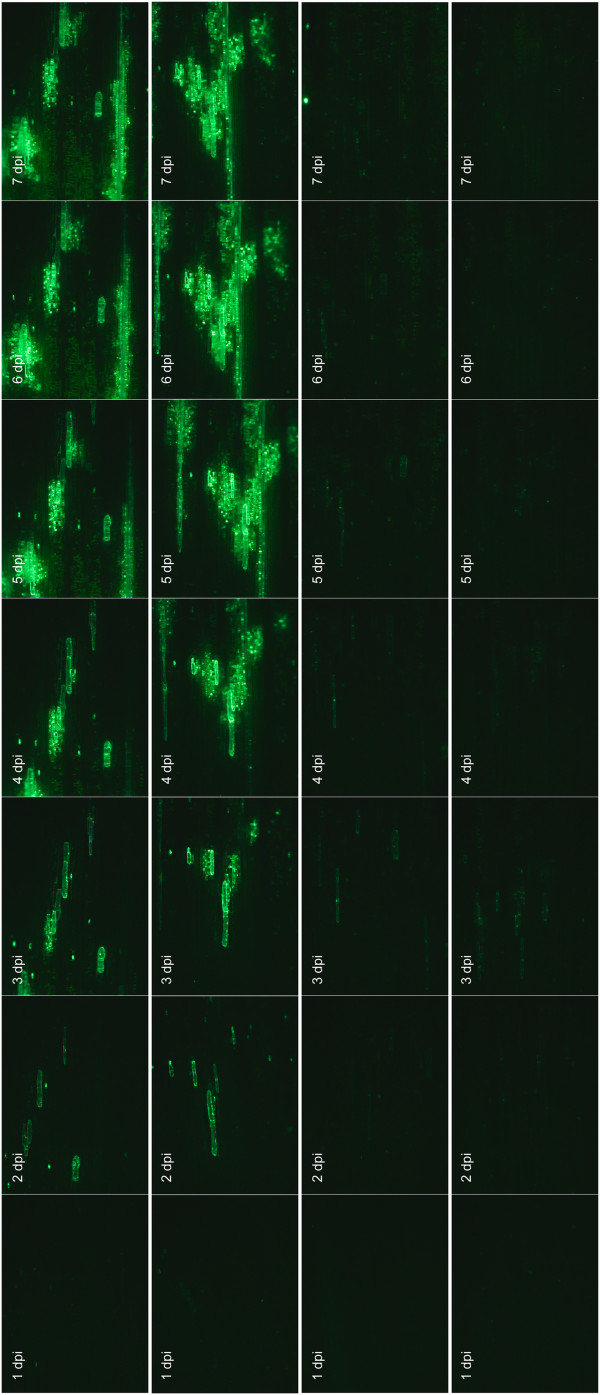
**1-7 days post inoculation of CP-EGFP and CPdelta-EGFP CfMV movement in inoculated oat leaves.** Upper two panels correspond to CP-EGFP and lower two to CPdelta-EGFP. Oat leaves were biolistically inoculated with mutant CfMV clones expressing EGFP fused to either the full length CP (CP-EGFP) or in place of the CP C-terminal region (CPdelta-EGFP). Virus localisation was monitored by EGFP fluorescence.

Oat plants were biolistically inoculated and virus movement was analysed by monitoring EGFP fluorescence in inoculated tissues. At 2 dpi the fluorescence was visible mainly in single epidermal cells and in small foci of mesophyll cells (Figure 7). The CP-EGFP fusion protein localised to the nucleus and cytoplasm of epidermal cells (data not shown). At 3 dpi the EGFP was detected in the neighbouring epidermal cells and the infection foci in the mesophyll were expanding. From the 4th to the 6th dpi the area of infected mesophyll cells continued to grow and presumably reached the vasculature. Beyond that time point the infected area stopped to grow and fluorescence started to fade.

In the case of CPdelta-EGFP the fluorescence was considerably weaker of what was observed with CP-EGFP. The fluorescence appeared strongest around the 3rd to the 4th dpi with CPdelta-EGFP (Figure 7). Weak fluorescence was detected in single epidermal cells after 2 dpi, which sometimes spread to neighbouring epidermal cells the following days. EGFP was not detected in mesophyll cells. The signal from epidermal cells usually disappeared around the 5th day (Figure 7). No distinguishable EGFP signal was detected in the upper leaves with either of the EGFP containing viruses.

### Detection of CfMV recombination mutants

**Figure 8:**
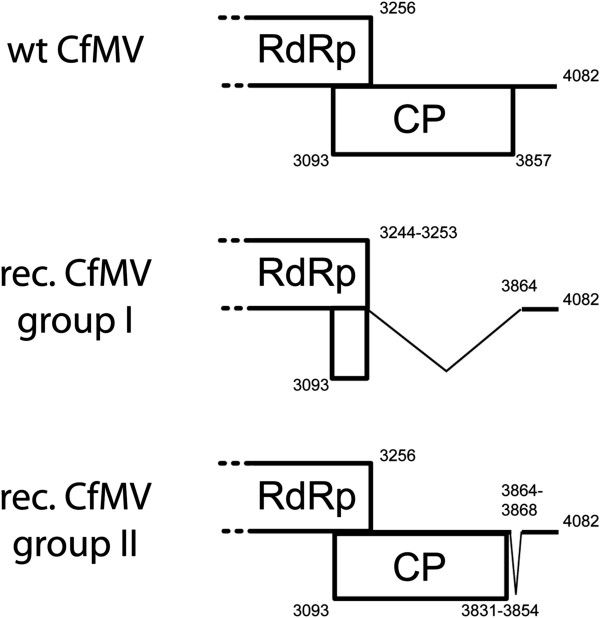
**Spontaneous deletion mutants from non-inoculated upper leaves of oat plants inoculated with CP-EGFP and CPdelta-EGFP.** Wt CfMV–the 3′ end organisation of CfMV genome. Rec. CfMV group I–group of recombinant viruses which had lost CfMV nt from 3244-3253 to 3864 of the RdRp and CP genes along with the EGFP sequence. Rec. CfMV group II–group of recombinants which had lost CfMV nt from 3831-3854 to 3864-3868 of the CP cistron together with EGFP sequence. The numbering corresponds to the nucleotides in wt CfMV genome.

Although no EGFP was detectable in the upper leaves of plants infected with CP-EGFP or CPdelta-EGFP, some of these plants developed CfMV infection symptoms in the upper leaves. This prompted for the further analysis of the infected and upper leaves. Indeed, viral RNA was detected from the upper leaves of inoculated plants by RT-PCR but the fragments had considerably lower molecular weight than expected. Sequencing revealed that the initially EGFP containing viruses had lost the EGFP sequence as well as different portions of CP/RdRp coding sequence (Figure 8). CfMV RdRp coding sequence ends at position 3256 and nucleotides (nt) 3093-3857 code the CP. Two different naturally occurring deletion mutant subsets were identified. In the first group nt from around 3244-3253 to 3864 of the RdRp and CP genes (numbering corresponds to wt CfMV) were deleted. Usually around 5 nt were present at the junction site, that could not be matched to CfMV sequence, but in some cases up to 65 nt of EGFP sequence were nested inside the CfMV sequence (data not shown). Deletion mutants belonging to the first group were detected in plants inoculated with either CP-EGFP or CPdelta-EGFP. The second group was comprised of viruses where nt between 3831-3854 to 3864-3868 at the end of the CP gene were missing. These mutants were obviously only detected in plants inoculated with CP-EGFP. Both deletion mutant groups were also detected in the inoculated leaves. With CPdelta-EGFP usually a single species were detected in one leaf, inoculated or systemic, whereas with mutant CP-EGFP usually several recombination mutant species were detected within the same sample, the largest in size (containing most of the CP sequence) being dominant (data not shown).

## Discussion

It was previously reported that P1 of CfMV is indispensable for virus movement and accumulation in oats [19] and that it is a suppressor of RNA silencing [20]. However, it still remains unclear whether P1 facilitates virus spread as an RNA silencing suppressor or as a member of the movement RNP complex. Here we demonstrate that CP is also an RNA silencing suppressor in 16c *N. benthamiana*. It is not a surprise that CfMV encodes two suppressors of RNA silencing, since more than one suppressor has been identified for other viruses as well [21,22]. Both suppressors, P1 and CP, are able to interfere with the RNA silencing mechanism independently and a strong synergistic effect was not observed. It seems plausible that CfMV CP contributes to virus spread through enhancement of accumulation. Such claim is supported by experiments with RYMV, which accumulated to higher levels in transgenic plants expressing RYMV CP compared to control plants, indicating that transgenic CP further enhanced virus infection and accumulation [23]. The authors speculated that CP may enhance viral accumulation by influencing replication or host susceptibility or, alternatively, by suppressing RNA silencing.

The arginine-rich region of the CP of sobemoviruses has been studied before. The CP of mutants R3L and R5X localises to cytoplasm and nucleus whereas the wt CP localises almost exclusively to the nucleus [13]. Results obtained here, with viruses containing the same mutations in CP, demonstrate that these mutations have no deleterious effect on virus cell-to-cell and systemic movement as well as on mechanical transmission in oats. The majority of CP-EGFP fusion protein localises to the cell nucleus when expressed independently from the rest of the virus genome [13]. Here we observed that when CP-EGFP was expressed together with the rest of the viral proteins, the fluorescence did not accumulate in the nucleus, but remained evenly distributed between cytoplasm and nucleus. Most probably the CP was interacting with other viral and/or host proteins or with full-length genomic RNA and therefore was not accumulating in the nucleus any more.

Despite these possible interactions, CfMV CP null mutant (noCP) was capable of cell-to-cell as well as systemic movement in all three tested hosts. The fact that CfMV CP is not strictly needed for the infection was further demonstrated by experiments with viruses expressing CP-EGFP and CPdelta-EGFP. As expected, the virus replicated in single epidermal cells and migrated to the mesophyll, beneath these epidermal cells, from where it presumably entered vascular tissue. Spontaneous CfMV recombination mutants detected in plants inoculated with mutants CP-EGFP/CPdelta-EGFP that lacked most of the CP cistron (group I, deleted nt 3254-3864), produced systemic infection as well as symptoms, again demonstrating the dispensability of CfMV CP for cell-to-cell and systemic movement.

In contrast, it has been previously documented that sobemoviruses require P1 as well as CP for systemic movement [14-17]. An RYMV CP mutant failed to infect rice plants systemically, but accumulated in the inoculated leaves, indicating cell-to-cell movement [14], whereas SCPMV CP initiation codon mutant, analogous to CfMV noCP, was undetectable even in inoculated leaves, but replicated in protoplasts [15]. The CP of *Turnip rosette virus,* another sobemovirus, has been reported to facilitate long distance movement of red clover necrotic mosaic dianthovirus [24]*.* Based on protein interaction studies of P1 with either CP or native virions of SeMV, closely related to SCPMV, Chowdhury and Savithri [18] have proposed a model for CP involvement in SeMV movement. We conclude that CfMV in general utilises a different movement strategy from SCPMV and RYMV.

Interestingly, we were unable to transmit noCP mechanically, whereas R3L and R5X were transmittable. This demonstrates that CP is necessary for an efficient sap-transmission and that mutations in the arginine-rich region do not affect the viral RNA-CP complex involved in this transmission. The mutant noCP should be, in theory, transmissible as well, since viral RNA is all what is needed for initiating the infection. Nevertheless, we have observed that mechanical inoculation with *in vitro* synthesised CfMV RNA is less efficient than biolistic inoculation with the same RNA (our unpublished observations). When compared to the R3L and R5X (that were transmissible), mutant noCP did not have significantly lower viral RNA levels in the systemically infected leaves which were used as the source for sap-inoculation (data not shown). Most likely the formation of virus particles is necessary for the efficient CfMV transmission.

Mutant R5X failed to produce systemic infection in mechanically inoculated plants. We propose that this deficiency is due to the mutation in RdRp yielding a less fit virus.

CPdelta-EGFP produced a considerably weaker EGFP signal in infected cells than CP-EGFP. It is possible that the EGFP sequence was just lost more rapidly than in case of CP-EGFP or that CPdelta-EGFP had considerably lower accumulation. It seems that, if available, the virus usually maintains as much of the CP sequence as possible. Foreign EGFP sequence seems to be the trigger for recombination because we have not encountered recombinant viruses in plants infected with mutant noCP or with the wt virus.

It is interesting that the mutants, which had lost the EGFP and CP coding sequences, all contained almost the entire 3′ UTR. The 3′ UTR starts at nt 3858 and the recombinant mutants had retained the sequence starting from nt 3865 or 3869. This leads us to speculate that the 3′ UTR might contain sequences or structural elements important for the transport, as the full-length 3′ UTR is not needed for replication, translation and accumulation in oats (Olspert, unpublished results). Another explanation is that nt 3865-3869 simply contain a hot-spot for recombination. These two hypotheses are, of course, not mutually exclusive.

## Conclusions

Sobemoviral P1 is known to be a suppressor of RNA silencing. This study demonstrates that, at least for CfMV, CP is a second suppressor.

The viral RNA genome is usually transported from cell-to-cell and systemically through the vasculature either as virions or as some other form of RNP complex. Trafficking as virions can be now ruled out for CfMV, at least in the tested host plants, since all the experiments reported here indicate that CP is not needed for cell-to-cell and systemic movement.

Altogether we have demonstrated that in different pathosystems individual sobemoviruses can exploit alternative cell-to-cell and long distance movement strategies. It would be interesting to determine whether the CfMV P1 RNA silencing suppressor activity can be uncoupled from the movement function and to determine the composition of the movement RNP complex. The RNA silencing suppression mechanism by which CP contributes to higher virus accumulation of CfMV deserves further investigation as well.

## Methods

### Construction of CfMV mutants

Base numbering of constructs generated for this study corresponds to CfMV Norwegian isolate [25]. All CfMV cDNA clones were created by modifying the original cDNA clone [19]. It was decided to simplify plant inoculation by removing the necessity for *in vitro* RNA synthesis before plant inoculation. To achieve this, the CfMV genome was cloned downstream of CaMV 35S promoter and *Hepatitis delta virus* ribozyme together with nopaline synthetase terminator were introduced after the genome in order to maintain the exact 3′ end of the genome after transcription. The exchange of the promoter and the addition of the ribozyme coupled with a terminator were carried out using overlap-extension-PCR with appropriate primers and standard cloning techniques.

cDNA clones of CfMV containing mutations were also generated using overlap-PCR. Mutations of R5X and R3L were introduced by primers described in Olspert et al. [13] and in mutant noCP the CP initiation sequence ATGATG was mutated to ACGACG. Using primers containing the mutations, the CfMV fragments containing nt 3096-3853 and 1604-3162 were produced and merged in a following round of PCR, so that a fragment corresponding to CfMV nt 1604-3853 was obtained. The latter fragment was used to introduce the mutations to the CfMV cDNA by employing *Nco*I sites at positions 2508 and 3619. In the case of virus mutant noCP, in addition to the CP initiation codon mutation a stop codon was introduced to CP reading frame after the overlap with RdRp gene (Figure 3B). This was achieved by cleaving the plasmid with *XmaJ*I restriction endonuclease at CfMV position 3311, filling in the termini and re-ligating the plasmid. This produced a reading frame switch starting from position 3311 and an in frame stop codon at position 3343.

Virus clones expressing CP-EGFP fusions were generated by replacing the CfMV sequence between *XmaJ*I and *Pst*I restriction sites at positions 3311 and 3869, respectively. In the case of CP-EGFP, the fusion sequence was obtained by PCR using a plasmid expressing the fusion protein [13] as the template. For CPdelta-EGFP EGFP primers with aforementioned restriction sites were used to generate the appropriate fragment.

All DNA constructs used in this work were verified by sequencing.

### Plant inoculation and virus detection

12-14 days old oat (cv. Jaak) plants were inoculated biolistically (Helios, BioRad) with CfMV constructs according to manufacturer’s instructions. Samples from the inoculated leaves were collected at 14 dpi and from upper leaves at 21 dpi. Each experiment was repeated at least twice and with a minimum of 8 plants per construct. Follow-up analysis in wheat (cv. Zebra) and barley (cv. Kymppi) were conducted once with 16 plants per construct. Plant tissue was homogenised with TissueLyzer (QIAGEN) and total RNA was extracted from samples according to Logemann et al. [26]. The analysis of RNA by RT-PCR was carried out with primers detecting nt 2749-4082 of the positive strand of viral RNA. The obtained RT-PCR fragments were purified from the agarose gel and the region containing the mutations was sequenced. Protein samples from the same material were obtained in parallel from the cell debris collected after the first centrifugation of RNA extraction. The pellet was suspended in PBS-Tween buffer and total protein was precipitated from the supernatant with trichloroacetic acid. Subsequently, the protein extract was analysed on 12.5% SDS-PAGE, blotted onto Hybond C membrane (GE Healthcare) and probed with rabbit polyclonal anti-CP antiserum [27]. Goat anti-rabbit HRP conjugate was used for detection. Ribulose-1,5-bisphosphate carboxylase oxygenase large subunit was visualised with Ponceau S (Sigma) stain for calibration.

For the analysis of mechanical transmission, the upper leaves of infected plants were ground in liquid nitrogen and the homogenate suspended in 10 volumes (w/v) of 100 mM potassium phosphate buffer (pH 7.0) containing 0.5% Celite. This suspension was used to mechanically inoculate plants and the following analysis of virus infection was done as in the case of bombarded plants.

### Agroinfiltration assay and siRNA analysis

CfMV CP coding sequence nt 3096–4082 was amplified with primers containing the appropriate restriction sites, excised with *Bam*HI and *Fsp*AI and cloned into pBin61 [28] between the 35S promoter and Nos terminator to give pBin61-CP. 35S-CP refers to *Agrobacterium tumefaciens* containing pBin61-CP.

The recombinant *A. tumefaciens* strain used throughout the experiment was C58C1 harbouring pCH32 [29]. Equal volumes of 35S-CP and 35S-GFP (*A. tumefaciens* carrying GFP, kindly provided by D. Baulcombe), as well as of 35S-P1 (*A. tumefaciens* containing CfMV P1 [20]) and 35S-GFP or 35S-P1 and 35S-CP together with 35S-GFP (total volume divided in three parts) were mixed and co-infiltrated (OD_600_ = 1) to *N. benthamiana* line 16c (kind gift of D. Baulcombe) leaves of 4-week-old plants, as described previously [30]. As a control, *A. tumefaciens* carrying the empty binary vector pBin61 was infiltrated together with 35S-GFP. Three independent experiments were carried out, each including 5-8 infiltrated plants for each mixture (35S-GFP + 35S-CP, 35S-GFP + 35S-P1, 35S-GFP + 35S-CP + 35S-P1 and 35S-GFP + pBin61). Infiltrated plants were kept in a plant chamber at 22°C under a 16-h photoperiod. GFP fluorescence was monitored using a hand-held 100 W, long-wave UV lamp (Black-Ray B-100AP, Ultraviolet Products) until 28 dpi. Plants were photographed with Pentax K200D digital camera and pictures were processed with Adobe Photoshop CS2 (version 9.0.2).

Total RNA was extracted from the infiltrated patches 7 dpi using TRIzol reagent (Invitrogen) according to the manufacturer’s instructions. For the analysis of GFP siRNA 20 μg of total RNA were denatured and loaded on 15% polyacrylamide gel (19:1 ratio of acrylamide to bis-acrylamide, 8 M urea). The gel was run at 400 V for 3.5 h and then cut in the middle. The lower half of the gel was transferred to Hybond N + -membrane by electroblotting in 0.5 X TBE buffer at 10 V overnight. ULTRAhyb-Oligo buffer (Ambion) was used for overnight hybridisation at 42°C. As a radioactive probe DNA oligo containing a sequence complementary to GFP (5′-CTCTTGAAGAAGTCGTGCCGCTTCATATGA-3′) was end-labeled with ^32^P by T4 polynucleotide kinase (Fermentas) and purified through NICK Sephadex G-50 columns (GE Healthcare) according to manufacturers’ protocols. The membrane was finally washed twice with 2X SSC, 0.1% SDS for 30 min at 42°C. As a reference marker we used a 30-nt [^32^P]-end labelled DNA oligo. As loading control we detected U6 snRNA in the same membrane with a [^32^P]-end labelled oligo according to Akbergenov et al. [31]. The Northern blot analysis of GFP mRNA was done as described above but using 5 μg of total RNA and 8% polyacrylamide gel. The hybridisation was carried out according to Szittya et al. [32] using as probe [^32^P]-labelled *in vitro* transcript corresponding to the anti-sense strand of GFP. The membrane was washed with greater stringency. Radioactive signals were detected using Personal Molecular Imager FX (BioRad) after 20 h (for siRNA detection) or 1 h exposure (for mRNA detection).

## Competing interests

The authors declare that they have no competing interests.

## Authors’ contributions

AO conceived the study, constructed CP mutants and carried out all experiments involving these mutants. KK performed the experiments involving EGFP expressing CfMV viruses. CS and JG carried out the RNA silencing suppression experiments. AO, KK and CS drafted the manuscript. ET conceived the study and helped to draft the manuscript. All authors read and approved the final manuscript.
